# FoodBase corpus: a new resource of annotated food entities

**DOI:** 10.1093/database/baz121

**Published:** 2019-11-04

**Authors:** Gorjan Popovski, Barbara Koroušić Seljak, Tome Eftimov

**Affiliations:** 1 Faculty of Computer Science and Engineering, Ss. Cyril and Methodius University, ul.Rudzer Boshkovikj 16, 1000 Skopje, Macedonia; 2 Jožef Stefan International Postgraduate School, Jamova cesta 39, 1000 Ljubljana, Slovenia; 3 Computer Systems Department, Jožef Stefan Institute, Jamova cesta 39, 1000 Ljubljana, Slovenia; 4 Department of Biomedical Data Science, Stanford University, 450 Serra Mall, Stanford 94305 CA, USA; 5 Center for Population Health Sciences, Stanford University, 450 Serra Mall, Stanford 94305 CA, USA

## Abstract

The existence of annotated text corpora is essential for the development of public health services and tools based on natural language processing (NLP) and text mining. Recently organized biomedical NLP shared tasks have provided annotated corpora related to different biomedical entities such as genes, phenotypes, drugs, diseases and chemical entities. These are needed to develop named-entity recognition (NER) models that are used for extracting entities from text and finding their relations. However, to the best of our knowledge, there are limited annotated corpora that provide information about food entities despite food and dietary management being an essential public health issue. Hence, we developed a new annotated corpus of food entities, named FoodBase. It was constructed using recipes extracted from Allrecipes, which is currently the largest food-focused social network. The recipes were selected from five categories: ‘Appetizers and Snacks’, ‘Breakfast and Lunch’, ‘Dessert’, ‘Dinner’ and ‘Drinks’. Semantic tags used for annotating food entities were selected from the Hansard corpus. To extract and annotate food entities, we applied a rule-based food NER method called FoodIE. Since FoodIE provides a weakly annotated corpus, by manually evaluating the obtained results on 1000 recipes, we created a gold standard of FoodBase. It consists of 12 844 food entity annotations describing 2105 unique food entities. Additionally, we provided a weakly annotated corpus on an additional 21 790 recipes. It consists of 274 053 food entity annotations, 13 079 of which are unique. The FoodBase corpus is necessary for developing corpus-based NER models for food science, as a new benchmark dataset for machine learning tasks such as multi-class classification, multi-label classification and hierarchical multi-label classification. FoodBase can be used for detecting semantic differences/similarities between food concepts, and after all we believe that it will open a new path for learning food embedding space that can be used in predictive studies.

## Introduction

In biomedical text mining, automation of information extraction (IE) aimed to uncover relations of any type from scientific literature has become a very important task. One of the first steps in IE is performed by named-entity recognition (NER) that locates named entities in the text to be classified into pre-defined categories. Best-performance NER methods are usually corpus-based (1–3), which require corpora of annotated entities of interest. Various annotated corpora have already been produced by shared tasks, such as BioNLP (4–8) and BioCreative (9–13), where the main aim is to challenge and encourage research teams on natural language processing (NLP) problems. These annotated corpora can be used for different research aims such as gene event extraction, cancer genetics, pathway curation, corpus annotation with gene regulation ontology, gene regulation networks in bacteria, bacteria biotopes, extracting the regulation of the seed development in plants, disease- and symptom-related entities, relations that exist between chemical/drug entities and disease entities, methods for annotations such as disease, phenotype, and adverse reactions in different text sources literature, family history information extraction, and clinical semantic textual similarity.

However, in 2019, Lancer Planetary Health published that 2019 is the year of nutrition, where the focus should be on discovering relations between food systems, human health, and the environment. Contrary to the large number of available annotated corpora with entities from the biomedical domain, in the food domain there are a limited number of resources that could be used for research.

Today, there are a vast number of recipes published on the internet, which carry valuable information about food and nutrition. However, to the best of our knowledge, there are only two existing corpora of annotated recipes: (i) the r-FG (recipe flow graph) corpus (14) and (ii) the CURD (Carnegie Mellon University Recipe Database) corpus (15). The r-FG corpus consists of 266 Japanese recipes annotated using eight tags related to *food*, *tool*, *duration*, *quantity*, *action by the chef*, *action by foods*, *state of foods* and *state of tools*. The CURD corpus consists of 300 annotated recipes and 350 unannotated ones, for which the Minimal Instruction Language for the Kitchen language (MILK) is used for annotation (15).

Let us mention the UCREL semantic analysis system (USAS), which is a framework for automated semantic analysis of text. It distinguishes between 21 major categories, one of which is also ‘food and farming’ (F) (16). Further, it provides additional semantic tag information that is used in the Hansard corpus (17). The Hansard corpus was recently created as part of the SAMUELS (Semantic Annotation and Mark-Up for Enhancing Lexical Searches) project (18), with the aim to extract speeches (i.e. digitised debates) given in the British Parliament from 1803 to 2005.

As part of our previous work (19–20), we developed drNER, which is a rule-based NER system used for IE from evidence-based dietary recommendations, where beside entities related to nutrition and dietary recommendations, food entities were also of our interest. However, drNER works with unstructured data. In drNER, food entities are extracted using the food semantic tags obtained by the UCREL semantic analysis on a token level combined with Boolean algebra rules in order to define phrases from text that are food entities.

Although abovementioned recipe-annotated corpora exist, they are limited. The r-FG corpus is composed only of Japanese food recipes, and both the r-FG corpus and the CURD corpus use annotation schemes that are not detailed enough, providing only a general food entity; without differing between groups of dishes (e.g. soups, grain dishes, egg dishes, tea, coffee). Also, drNER provides only a general food entity because it was developed to distinguish between *food*, *nutrient* and *quantity/unit*. The USAS can provide additional information about the selected food entity, but its limitation is that it works on a token level. A token, as defined as a problem in NLP, is a string of contiguous characters between pre-defined delimiters (e.g. white spaces, punctuation). Most commonly, a single token is a single word, number or abbreviation. For example, if we have ‘grilled chicken’ as one food entity that needs to be processed for its relations, the entities ‘grilled’ and ‘chicken’ will obtain separate semantic tags. For these reasons, we decided to create a FoodBase, which is a new corpus that can be used for automated food named-entity extraction and includes food entities annotated with the semantic tags from the Hansard corpus.

## Methods and Materials

In this section, we present how a resource of recipes to be used for IE was selected. Then, the Hansard corpus of semantic tags is described in more detail. We continue by presenting FoodIE, i.e. a rule-based NER (21), that is used for structuring recipes. First, we briefly describe its basic steps and then we focus on its evaluation and the introduction of a new step that was added to FoodIE with the aim of the semantic annotation of the extracted food entities.

### Recipe selection

To start creating the FoodBase corpus with annotated food entities, we selected 1000 various recipes from Allrecipes (22), which is the largest food-focused social network where everyone plays a part in helping cooks discover and share the home cooking. We selected this network because everyone can post recipes on Allrecipes, so we have variability in how users express themselves. The recipes were selected from five recipe categories: ‘Appetizers/Snacks’, ‘Breakfast/Lunch’, ‘Dessert’, ‘Dinner’ and ‘Drinks’, including 200 recipes for each recipe category. For each recipe, we collected information about the English recipe name, its ingredient list and the preparation instructions in English. The ingredient list consisted of English ingredient names and quantities in non-standard units and household measures provided in English (e.g. ‘1 large eggplant, halved lengthwise’, ‘1 (8 ounce) package crumbled feta cheese’).

### Hansard corpus semantic tags

In order to annotate food entities extracted from the selected recipes, we used semantic tags from the Hansard corpus (17). In this corpus, semantic tags are ordered using a hierarchical structure, where food is addressed in the category ‘Food and drink’ (AG). The AG category is further split into three subcategories: ‘Food’ (AG:01), ‘Production of food, farming’ (AG:02) and ‘Acquisition of animals for food, hunting’ (AG:03). The ‘Food’ subcategory consists of 125 top level semantic tags, the ‘Production of food, farming’ consists of 36 top level semantic tags and the ‘Acquisition of animals for food, hunting’ consists of top level 13 semantic tags. In addition to the AG category, we decided to also use the categories ‘Animals’ (AE) and ‘Plants’ (AF), so that any missing information (semantic tag) for a food entity that is a recipe ingredient could be searched for in AE and AF, as part of nature animal or plant, respectively. The AE category consists of 15 semantic tags, while the AF category consists of 30 semantic tags. There are additional and more specific tags on a deeper hierarchical level within some of these tags, which are also utilised. More details about the Hansard corpus semantic tags can be found in Hansard (17).

### FoodIE: a rule-based food NER

To enable NER that locates food entities, we have recently proposed a rule-based approach, called FoodIE, which works with unstructured textual data (i.e. recipe description) and consists of four steps (21):

1. **Food-related text pre-processing:** one of the main concerns of this step is to clean up the raw textual data, such as removing non-standard characters, excess white spaces and performing transliteration as to not confuse the taggers.
2. **Text POS-tagging and post-processing the tag dataset:** this step consists of acquiring the textual data with Part of Speech tags, as well as ensembling both taggers’ data to increase robustness.
3. **Semantic annotation of food tokens in the text:** this is the main rule engine of FoodIE, which utilizes a small number of rules and performs semantic annotation of the tokens in the text, classifying it in one of four classes which are further used to perform NER.
4. **Food name-entity recognition:** this step is concerned with chaining the semantically annotated tokens into food chunks that represent a single food concept.

For the aims of creating the FoodBase corpus, we added an additional step to the end of the FoodIE pipeline:

1. **Semantic annotation of the extracted food entities**: here, the Hansard semantic tags are grouped within each token for each food chunk, with the goal of representing the food concept in its entirety.

**Figure 1:**
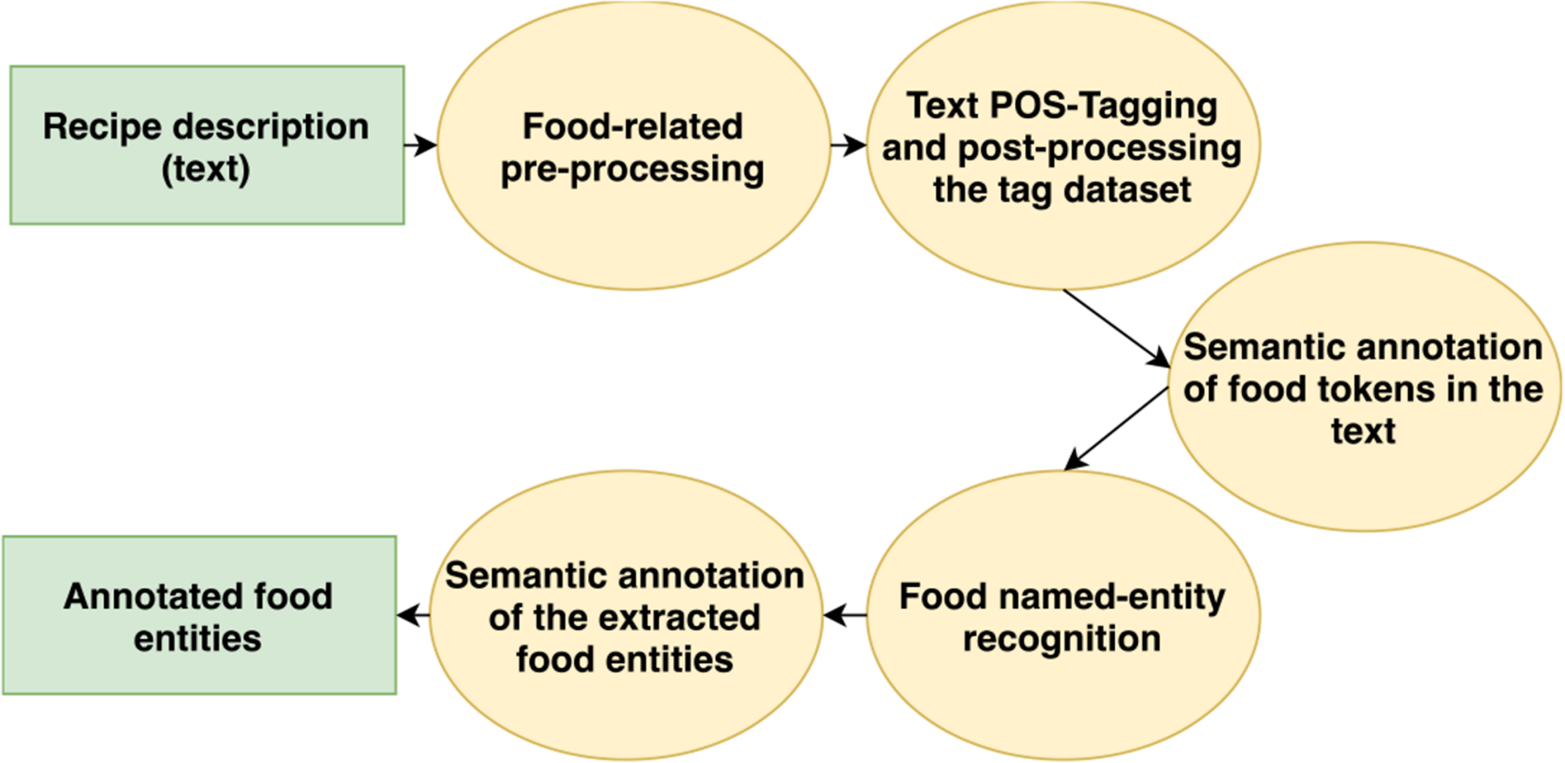
Flowchart of the extended FoodIE methodology.

The flowchart of the extended methodology is presented in Figure 1. More details about the first four steps have already been presented in our previous work (21); however, in this paper, we will focus on the evaluation of FoodIE as this is the crucial step in building the annotated corpus. An example of running FoodIE on one recipe is explained in (21), step by step. Then, we will describe the new step of semantic annotation of the extracted food entities.

#### Evaluation of the extended FoodIE methodology

Once the information about the recipes was selected from Allrecipes, we asked a person to manually extract food chunks from the description of each recipe. A food chunk is a contiguous collection of tokens which describe a single food concept. Then, we run the first two steps of FoodIE to obtain automatically extracted food chunks from the description of the same recipes. To avoid any kind of bias when comparing the food chunks extracted manually and automatically by FoodIE, another person was asked to cross reference the manually obtained chunks with the ones obtained by FoodIE. Using this method, true positives (TPs), false positives (FPs) and false negatives (FNs) were counted, while it was decided that the category true negative (TN) is not applicable to the nature of the problem and its evaluation. In our case, TP and FP mean outcomes where FoodIE correctly or incorrectly predicted the positive class, respectively. Similarly, a FN means an outcome where FoodIE incorrectly predicted the negative class. In addition to the results for TP, FP and FN, the results for ‘Partial (Inconclusive)’ are presented. This group of outcomes includes evaluations that could be either TP or FP/FN. For example, in the text segments ‘empty passion fruit juice’, ‘cinnamon’ and ‘soda,’ the actual food entity chunks are ‘passion fruit juice’, ‘cinnamon sticks’ and ‘club soda’, respectively. These occurrences are mostly due to the dual nature of words, meaning that a word is a synonym for both a noun and a verb or a synonym for an adjective and a verb. For such words, the FoodIE tagger sometimes incorrectly classifies the tokens. In these examples, ‘empty’ is tagged as an adjective, where in context it is a verb. The explanation is almost identical for the other two examples. For these reasons, when the evaluation metrics were being calculated, the ‘Partial (Inconclusive)’ category was omitted. Moreover, even if they are classified either as TP or as FP/FN, they would not significantly affect the results. We performed this kind of the evaluation in our previous study (21) since there is no pre-existing method to evaluate such a text corpus.

**Checking the concept.** First, a subset of 200 recipes out of 1000 were processed and evaluated. From each category, we selected 40 recipes. More details about the predictions are presented in (21).

Most of the FNs are related to food concepts that are represented by their brand names (e.g. ‘Snickers’, ‘Jim Beam’). Some of them also occur when the semantic tagger incorrectly classifies some token with regard to the context in which they are mentioned (e.g. ‘date’ classified as a day of year, when it represents fruit). Furthermore, there are also examples with some specific foods related to some cultures (e.g. ‘kefir’).

In the case of FPs, most of the instances are related to concepts related to food, but not food concepts by themselves. In most cases, these are instruments or tools used in cooking.

**Second trial.** Once the effectiveness of the concept was evaluated on 200 recipes, the complete set of 1000 recipes was processed and evaluated, and predictions for them are presented in (21).

Comparing the evaluation metrics for 200 and 1000 recipes presented in (21), we can conclude that FoodIE behaves consistently. Evaluating the dataset with 200 recipes, which consists of 100 recipes that were analysed to build the rule engine and 100 new recipes that were not analysed beforehand, we obtained a precision of 0.9761, a recall of 0.9430 and a *F*_1_ score of 0.9593. Furthermore, by evaluating it on the dataset of 1000 new recipes, we obtained 0.9780 for precision, 0.9437 for recall and 0.9605 for the *F*_1_ score. From these results, we can conclude that FoodIE gives very promising and consistent results.

#### Semantic annotation of the extracted food entities

Once food entities were extracted using FoodIE, we annotated each of them using the semantic tags provided by the Hansard corpus. For this reason, annotations that are assigned to each food chunk are the semantic tags that belong to the tokens from which the chunk is constructed. As we explained before, these tags come only from three general Hansard corpus categories, i.e. ‘Food and drink’ (AG), ‘Animals’ (AE) and ‘Plants’ (AF). When a selected entity recognized as a food entity cannot be annotated with any semantic tag from the ‘Food and drink category’, a tag from either ‘Animals’ or ‘Plants’ is used. Moreover, when no semantic tag can be associated to the food entity, it is assigned to the top food level hierarchy, i.e. ‘AG.01[Food]’.

Examples include the following:

- ‘grilled chicken’ obtains the semantic tags AG.01.t.07[Cooking] /AG.01.d.06[Fowls]
- ‘tortilla chips’ obtains AG.01.n.11[Bread] /AG.01.n.12[Pancake/tortilla/oatcake]
- ‘dry ranch salad dressing mix’ obtains AG.01.h.02 [Vegetables]/AG.01.m [Substances for food preparation]/AG.01.n.09 [Prepared vegetables and dishes]
- ‘cauliflower’ obtains AG.01.h.02.d [Cabbage/kale]

**Manual evaluation**. Semantic annotations obtained by FoodIE were manually evaluated. Food entities reported as FPs were manually excluded from the corpus, while the food entities reported as FNs were included in the corpus. This was done in order to obtain a good benchmarking dataset, which contains all food entities that are present in the dataset of 1000 randomly selected recipes from five main dish categories. Furthermore, apart from excluding FPs and including FNs, the annotated semantic tags were double-checked. During this process, all the incorrect semantic tags were removed, while all the missing semantic tags were added to specific food entities.

**Figure 2:**
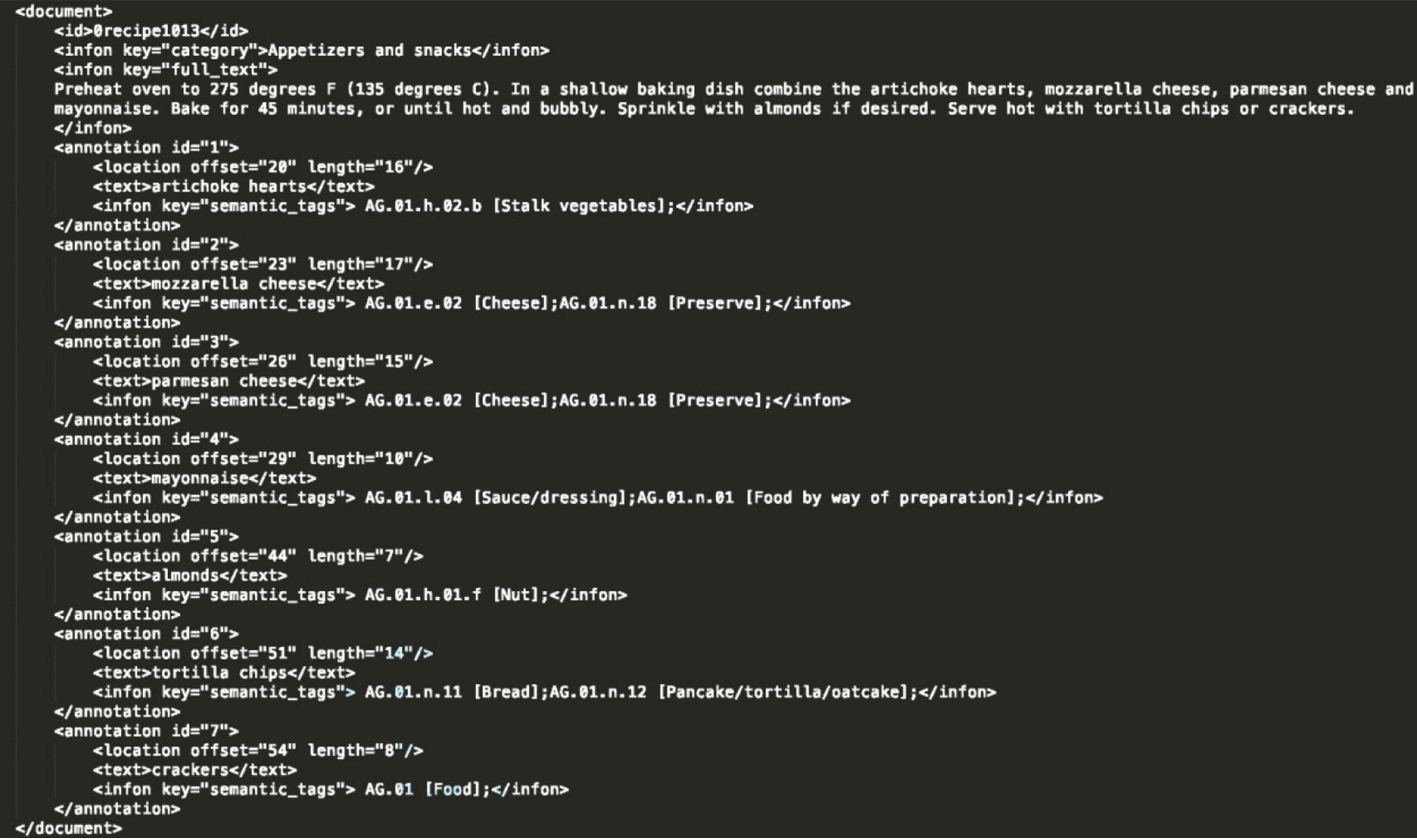
Annotated recipe from ‘Appetizers and snacks’ category presented in the BioC format. For the recipe presented in this figure, all the extracted food concepts are presented, along with their respective semantic tags and their location in the raw recipe text.

**Annotation format.** We decided to annotate the extracted information using the BioC format (23), which has been originally proposed by biomedical NLP and text mining tools. It is a simple XML-based format aimed for sharing text data and annotations, with the goals of simplicity, interoperability and broad use and reuse. In Figure 2, a selected recipe is presented in the BioC format.

**Post-processing of the annotated semantic tags.** While manually evaluating and correcting the semantic annotations generated by FoodIE, the food entities reported as FNs were incorporated into the FoodIE rule engine as a resource, with the goal to improve its performance. This means that the new version of the FoodIE rule engine is more robust, since it does not incorrectly produce the FNs that were manually added as a resource. In addition to this, there were some specific instances where the semantic tags themselves needed a modification in some way. For example, the semantic tag ‘AG.01.af [Tea manufacture]’ incorrectly appears every time the token ‘mixture’ is present in the food entity chunk, so it is removed from the list of semantic tags for that food chunk. Another example of this is the semantic tag ‘AG.01.ae.03 [Brewing]’ that incorrectly appears whenever the token ‘mashed’ is present. If the food entity does not contain any semantic meaning relevant to these semantic tags, the tag is removed. In addition to this, some semantic tags were very vague. The semantic tagger occasionally did not tag the food entity ‘water’ as such, but just provided the tag ‘AG.01 [Food]’. In such cases, the tag ‘AG.01.z [Water]’ was manually added for that food entity, while the original one was removed. Such omissions and inclusions, although rare (<5%), were performed as needed.

**Justification of using semantic tags from Hansard corpus.** Before we decided which semantic tags will be used to describe the extracted food entities, we tried several other knowledge resources for identifying food entities. We did this by using the set of 1000 recipes. To extract and describe the food entities, we used two approaches: FoodIE and NCBO annotator (24). The NCBO Annotator is a web service that annotates text provided by the user by using relevant ontology concepts. It is available as part of the BioPortal software services (28). The annotation workflow is based on an efficient syntactic concept recognition engine (which utilizes concept names and synonyms), as well as on a set of semantic expansion algorithms that leverage the semantic information found in ontologies. The methodology relies on ontologies to create annotations for textual data and presents them by using semantic web standards. It can be also used for named-entity extraction from food ontologies that are part of the BioPortal software services. FoodIE annotates the food entities using the semantic tags from the Hansard corpus, while the NCBO annotator was used for annotation in a combination with three food ontologies that are available in the BioPortal (i.e. FoodOn (25), OntoFood and SNOMED CT (26)). We should mention here that SNOMED CT is also part of the Unified Medical Language Systems (UMLS) (27). Each version of the NCBO annotator working with a different ontology was assumed to be as a different NER method (i.e. NCBO (SNOMED CT), NCBO (OF), NCBO (FoodOn)). Finally, a total of four different NER methods (FoodIE, NCBO (SNOMED CT), NCBO (OF) and NCBO (FoodOn)) that can be used for food information extraction were compared.

To evaluate the results, we selected three standard types of matches: true positives (TPs), false negatives (FNs) and false positives (FPs), as well as the aforementioned ‘Partial (Inconclusive)’ match type. The results from counting the instances of each match type are presented in Table 1. It is important to note that not all ontologies provided annotations for each recipe. More specifically, out of 1000 recipes, SNOMED CT missed 6, OntoFood missed 71, and FoodON missed 5. Next, we are going to explain the results for every match.

**Table 1:** Results from comparing different NER methods in the food domain

	FoodIE	SNOMED CT	OF	FoodOn
TPs	11 461	5100	2279	5725
FPs	258	472	378	1502
FNs	684	5327	9026	4968
Partials	359	2705	1591	2365

Looking at the comparison results in Table 1, we can see that the number of TPs is substantially larger when using FoodIE (11461) when compared to the three other ontologies with the NCBO annotator, i.e. SNOMED CT (5100), OF (2279) and FoodON (5725). The number of TPs should be maximized.

Moving on to the FPs, FoodIE again provides the best results of the four, while FoodON provides significantly more FPs than the other three NER methods. Respectively, they provide FoodIE (258), SNOMED CT (472), OF (378) and FoodON (1502). The number of FPs should be minimized.

The last of the standard types of matches is FN, where FoodIE once again behaves superiorly to the other three NER methods. The numbers here are FoodIE (684), SNOMED CT (5327), OF (9026) and FoodON (4968). The number of FNs should be minimized.

The last type of match we take into account is the partial match type. It is not clear whether this type of match should be maximized or minimized in and of itself, as it heavily depends on the number of other types of matches (especially TPs and FNs). For example, ideally all the food concepts would be matched as TPs and none as FNs. However, if a TP match is not encountered for a specific food concept instance, the second-best occurrence would be to ‘partially’ match it. The worst-case scenario is when the food concept is matched as a FN.

By performing the analysis, we can conclude that FoodIE, using the Hansard corpus, provides the most promising results since it can extract a larger number of food concepts as opposed to the NCBO annotator in combination with the selected ontologies. Moreover, the results imply that the three food ontologies (i.e. SNOMED CT, FoodOn, OntoFood) do not represent the food domain exhaustively, as many food concepts are not extracted using the NCBO annotator running on these ontologies. This indicates that they do not exist as entities in the food ontologies themselves.

**Food ontologies alignment.** To align food concepts in different food ontologies, we have created a resource, named FoodOntoMap, that consists of food concepts extracted from recipes. For each food concept, semantic tags from four food ontologies are assigned. With this, we create a resource that provides a link between different food ontologies, which can further be reused to develop applications for understanding the relation between food systems, human health and the environment.

**Table 2:** Descriptive statistics for the number of words per recipe, the number of food entities per recipe and the number of semantic tags per food entity

	**Number of words per recipe**
	**Curated**	**Un-curated**
Mean	106.40	114.78
Median	99.00	106.00
Mode	121.00	91.00
Standard Deviation	64.44	67.61
	**Number of food entities per recipe**
	**Curated**	**Un-curated**
Mean	12.85	12.58
Median	12.00	12.00
Mode	5.00	10.00
Standard deviation	7.22	6.71
	**Number of semantic tags per food entity**
	**Curated**	**Un-curated**
Mean	1.74	1.87
Median	2.00	2.00
Mode	1.00	1.00
Standard deviation	0.91	1.01

The results from FoodOntoMap are four different datasets and one data set mapping. Each dataset consists of an artificial ID for each unique food concept that is extracted by using each approach, the name of the extracted food concept and the semantic tags assigned to it. Each dataset corresponds to one of the four semantic resources: Hansard corpus, FoodOn, OntoFood and SNOMED CT. At the end, there is one data set mapping, called FoodOntoMap, where for each concept that appears at least in two datasets, the mapping between them is provided by listing the artificial ID of the concepts from each of the datasets in which it is encountered. The datasets consist of 13 205, 1069, 111 and 582 unique food concepts, obtained using Hansard corpus, FoodOn, OntoFood and SNOMED CT, respectively. The FoodOntoMap mapping consists of 1459 food concepts that are found in at least two of the food semantic resources.

The motivation for building such a resource in the food domain comes from the existence of the UMLS, which is extensively used in the biomedical domain. For example, the MRCONSO.RRF table that is a part of the UMLS is used in a lot of semantic web applications since it can map the medical concepts to a variety of different biomedical standards and vocabularies.

## Results and Discussion

### FoodBase corpus overview

After applying FoodIE for semantic annotation of 1000 randomly selected recipes with semantic tags from the Hansard corpus and performing post-processing of the annotated semantic tags, the initial FoodBase corpus was generated. It consists of 12 844 food entities extracted from the selected recipes for dishes from five main groups, with 2105 unique food entities in total.

Because the evaluation of extended FoodIE gave very promising and consistent results, we used it again for extracting and annotating food entities for a new, more extensive subset of 21 790 recipes from the same five groups of dishes, i.e. ‘Appetizers/Snacks’, ‘Breakfast/Lunch’, ‘Dessert’, ‘Dinner’ and ‘Drinks’. The outcome was the next version of the FoodBase corpus that includes much more recipes and corresponding food entities (274 053 total food entities, with 13 079 unique food entities). However, this version has not been processed manually to exclude the FPs and to include the FNs. To distinguish between the two versions of the FoodBase corpus, we call the manually post-processed version containing 1000 recipes ‘curated’ and the one consisting of 21 790 recipes as annotated by FoodIE, ‘un-curated’.

**Figure 3:**
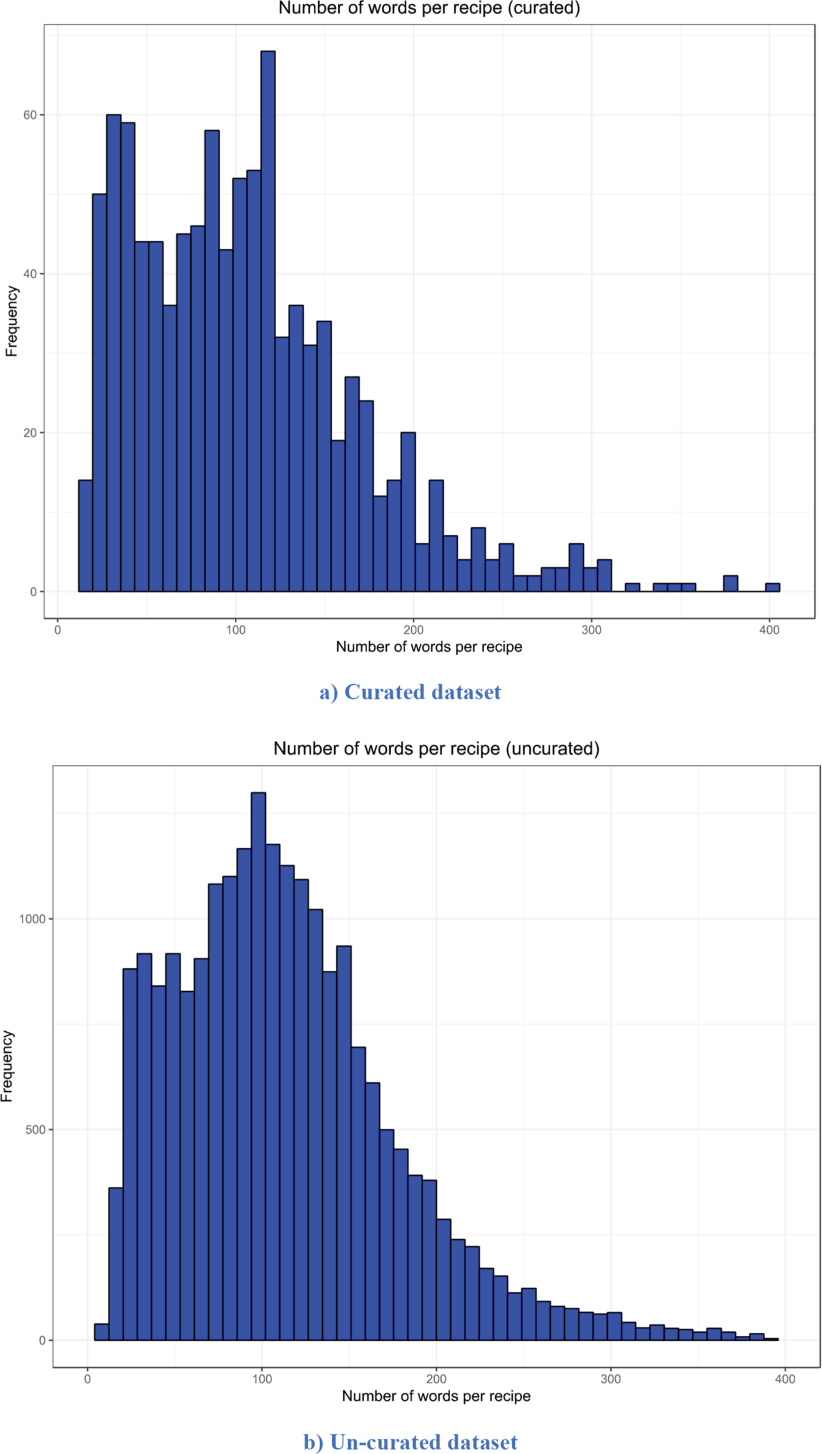
Distribution of the number of words per recipe. It is apparent that both distributions have a similar trend. However, the distribution of the un-curated version is much smoother because it includes more recipes. (a) Curated dataset. (b) Un-curated dataset.

**Figure 4:**
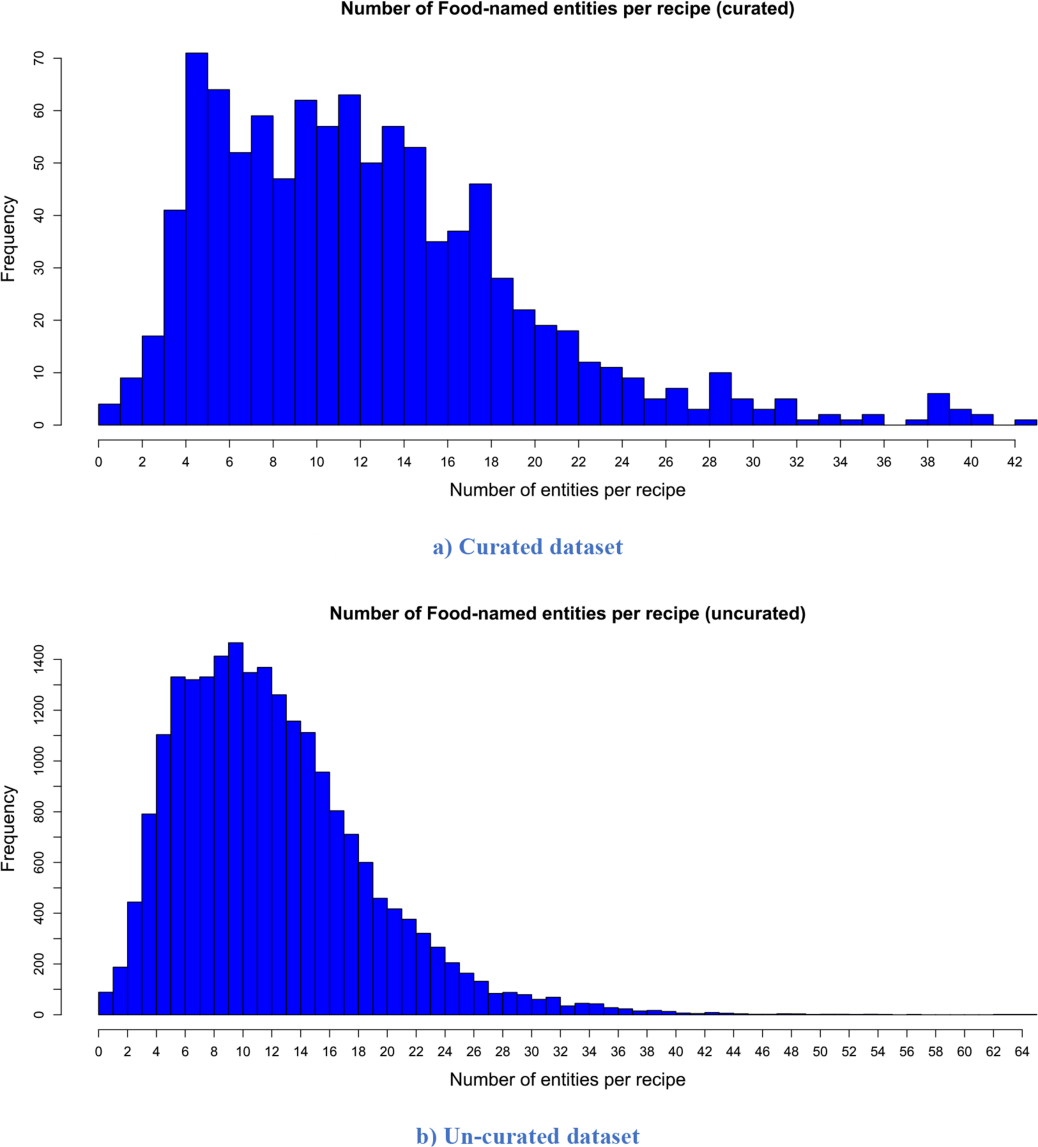
Distribution of the number of extracted food entities per recipe. It is apparent that both distributions have a similar trend. However, the distribution of the un-curated version is much smoother because it consists of more recipes. (a) Curated dataset. (b) Un-curated dataset.

**Figure 5:**
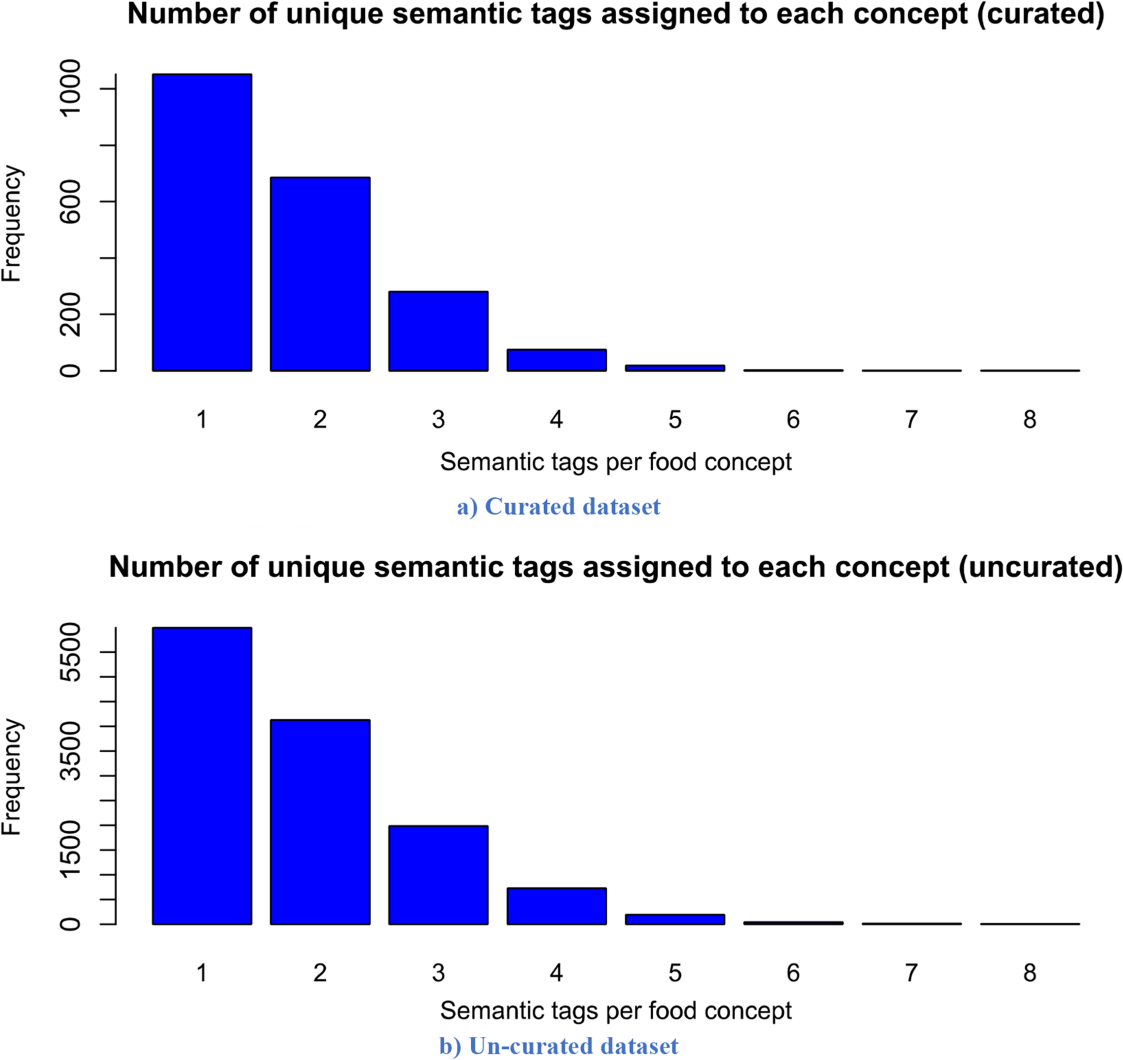
Distribution of the number of assigned semantic tags per food entity. Analysing it, it follows that both distributions have a similar trend, which is a power law distribution.

The descriptive statistics (i.e. mean, median, mode and standard deviation) for the number of words per recipe, the number of entities extracted per recipe and the number of semantic tags assigned per food entity for both versions are presented in Table 2. The distribution of the number of words per recipe for the curated and un-curated version of FoodBase is presented in Figure 3, while the distribution of the number of food entities extracted per recipe is presented in Figure 4. Additionally, the distribution of the number of semantic tags assigned per food entity for both versions is presented in Figure 5.

Looking at the descriptive statistics provided for the number of words per recipe, it is apparent that there are no big differences between the descriptive statistics of both versions. The biggest difference appears for the mode. The mode for the curated version is 121.00, while that for the un-curated version is 91.00. If we look more closely at the distribution provided for the curated version in Figure 3, we can see that these two values for frequency are close and that they differ by no more than 15. Moreover, it follows that the distributions have a similar trend. However, the distribution of the un-curated version is much smoother because it includes more recipes. The same conclusion is also true for the descriptive statistics provided for the number of extracted food entities per recipe. The only difference that is apparent is for the modes. It is 5.00 for the curated version and 10.00 for the un-curated version. However, if we look at the distribution of the curated version (Figure 4), we can see that the difference between their frequency is less than 10. From Figure 4, it is obvious that the distributions have a similar trend, the only difference is that the distribution of the un-curated version is much smoother, which is reasonable, since it includes more recipes. In Figure 5, the distribution of the number of assigned semantic tags per food entity is presented for both FoodBase versions, separately. Analysing it, it follows that both distributions have a similar trend, which is a power law distribution.

Additionally, the statistics are presented for each category ‘Appetizers/Snacks’, ‘Breakfast/Lunch’, ‘Dessert’, ‘Dinner’ and ‘Drinks’ separately in Table 3. It is evident that in both versions of FoodBase the average number of extracted entities, as well as the standard deviation, is the largest in the ‘Dinner’ category. There is no big difference between the average number of extracted entities from the ‘Appetizers/Snacks’, ‘Breakfast/Lunch’ and ‘Dessert’, and additionally, they have similar standard deviations. The ‘Drinks’ category has the smallest average number and standard deviation of extracted entities. If we compare the descriptive statistics for each category between both versions, we can see that there are not big deviations between their values.

**Table 3:** Descriptive statistics for the number of food entities per recipe and the number of semantic tags per food entity, for each category separately

**Number of food entities per recipe**								
		**Curated**				**Un-curated**	
	Mean	Median	Mode	Sd	Mean	Median	Mode	Sd
Appetizers/snacks	11.57	10.00	8.00	6.21	11.25	10.00	7.00	6.20
Breakfast/lunch	13.46	13.00	13.00	6.32	13.04	12.00	11.00	6.33
Dessert	15.15	14.00	15.00	7.03	13.79	13.00	12.00	6.54
Dinner	17.38	16.00	11.00	7.43	17.50	16.00	15.00	8.03
Drinks	6.67	6.00	5.00	3.53	6.52	6.00	6.00	3.31
**Number of semantic tags per food entity**								
		**Curated**				**Un-curated**	
	Mean	Median	Mode	Sd	Mean	Median	Mode	Sd
Appetizers/Snacks	1.59	1.00	1.00	0.77	1.69	1.00	1.00	0.88
Breakfast/Lunch	1.58	1.00	1.00	0.75	1.75	2.00	1.00	0.89
Dessert	1.71	1.00	1.00	0.90	1.88	2.00	1.00	1.01
Dinner	1.65	1.00	1.00	0.84	1.72	2.00	1.00	0.86
Drinks	1.82	1.00	1.00	1.02	1.96	2.00	1.00	1.14

**Figure 6:**
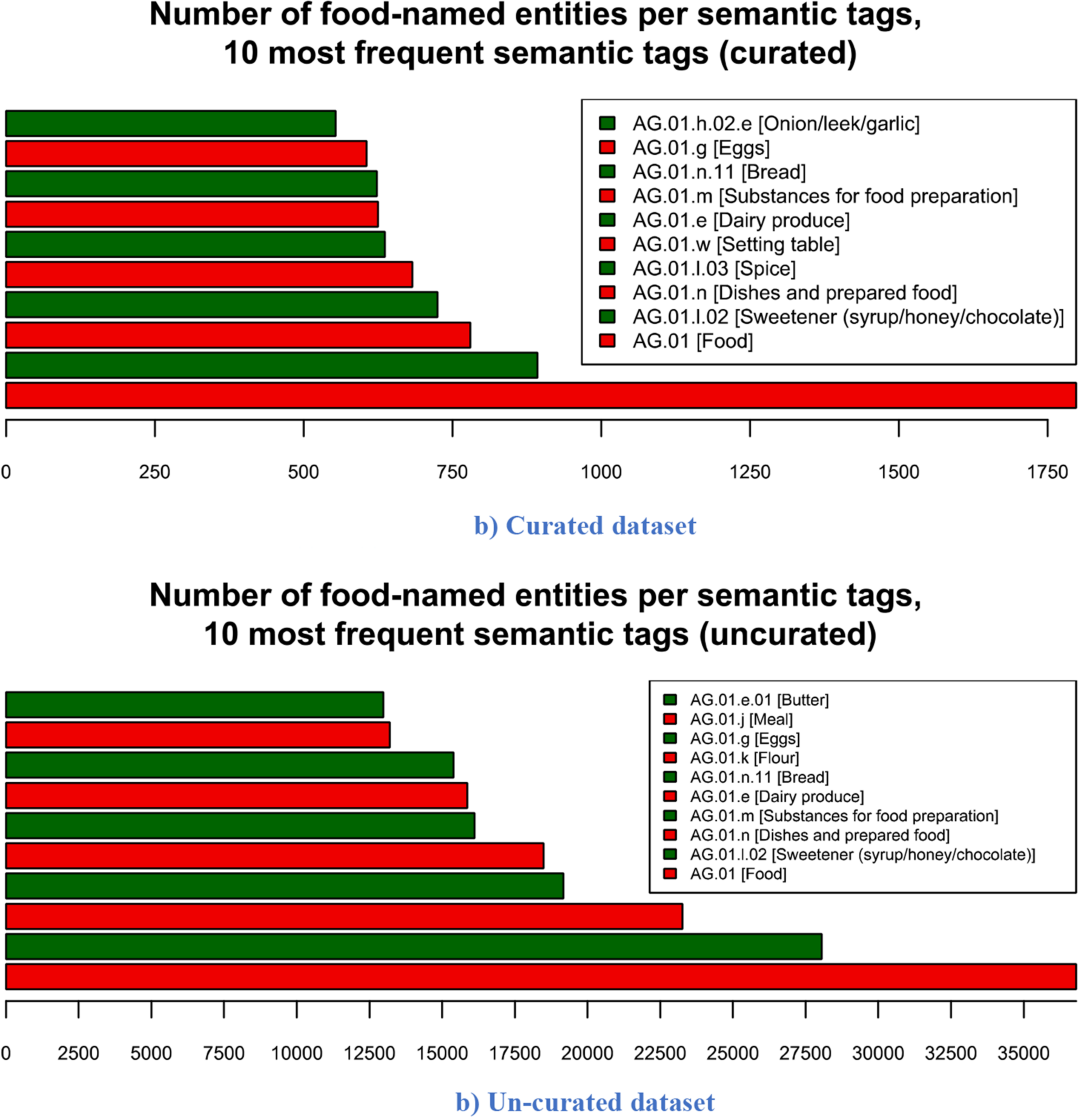
Number of food-named entities per 10 most frequent semantic tags. From the 10 most frequent semantic tags in both the curated and un-curated version, 7 are identical across both versions. The three that differ are due to the difference in the number of recipes in both versions. (a) Curated dataset. **(**b) Un-curated dataset.

The number of food entities per the 10 most frequent semantic tags for both FoodBase versions is presented in Figure 6. The most frequent tag for both versions is the ‘AG.01 [Food]’, which is the top level in the food category hierarchy in the Hansard corpus. This result comes from the fact that if there is no semantic tag assigned to the entity, but it is recognized as food, then it is automatically assigned to this semantic tag. If we compare the other nine most frequent semantic tags, we can see that the semantic tags ‘AG.01.l.02 [Sweetener (syrup/honey/chocolate)]’, ‘AG.01.m [Substance for food preparation]’, ‘AG.01.n [Dishes and prepared food]’, ‘AG.01.e [Dairy products]’, ‘AG.01.n.11 [Bread]’ and ‘AG.01.g [Eggs]’ appear in both versions of FoodBase. The initial FoodBase based on 1000 recipes also includes ‘AG.01.l.03 [Spice]’, ‘AG.01.w [Setting table]’ and ‘AG.01.h.02.e [Onion/leek.garlic]’, while the next FoodBase version based on 21 790 recipes includes ‘AG.01.k [Flour]’, ‘AG.01.j [Meal]’ and ‘AG.01.e.01 [Butter]’.

**Table 4:** Availability of all data and tools related to the FoodBase resource

**Resource/tool name**	**Availability**
FoodBase	http://cs.ijs.si/repository/FoodBase/foodbase.zip
FoodIE	https://github.com/GorjanP/FOM_mapper_client
FoodOn	https://foodontology.github.io/foodon/
OntoFood (OF)	https://bioportal.bioontology.org/ontologies/OF/?p=summary
SNOMED CT	https://confluence.ihtsdotools.org/display/DOC/Technical+Resources
Hansard corpus	https://www.hansard-corpus.org/
NCBO annotator	http://bioportal.bioontology.org/annotator
NCBO annotator REST API	http://data.bioontology.org/documentation
FoodOntoMap	https://doi.org/10.5281/zenodo.2635437

Looking at the 10 most frequent tags for both versions, we can see that 7 out of 10 semantic tags are the same. There is a difference between the frequencies of the semantic tags in both versions, since the un-curated version consists of more recipes. However, the first idea of building such a corpus is that it can be generally used for the bi-classification problem (food vs. not food concept), where the semantic tags are not crucial. Further, using some sampling techniques, different subsets can be generated from the un-curated version with regard to the semantic tags that will be of interest (e.g. top 5 or 10 most frequent) in order to produce more representative data sets for training corpus-based NERs.

In general, the difference between the curated and un-curated versions is that the un-curated version consists of FPs, which in most of the cases are related to objects that are not food concepts but are concepts closely related to food entities or an instrument for food or cooking. Also, the FNs are not included, and they are related to food entities that are branded food products, or some rare foods that are typical for some cultures.

The availability of data and tools used to create FoodBase corpus is provided in Table 4.

## Conclusions

Our motivation to start building the FoodBase corpus has been to provide the scientific community with a fundamental resource required for learning corpus-based methods that can be used for food-named entity recognition. FoodBase is presented in two versions: curated and un-curated. The curated version is manually evaluated, consisting of 1000 recipes, while the un-curated version consists of 21 790 recipes. For this reason, we can consider FoodBase as a whole as a ‘silver standard’. It can be also used as a benchmark dataset for several ML tasks, such as multi-class classification (29), multi-label classification (30) and hierarchical multi-label classification (31). Multi-class classification is applied when a food entity may be annotated with several semantic tags (e.g. ‘Food’ (AG:01); ‘Production of food, farming’ (AG:02); and ‘Acquisition of animals for food, hunting’ (AG:03)). Multi-label classification is performed when an output is a more complex structure such as a vector of tags with some dependencies among them (i.e. the food entity can belong to multiple semantic tags simultaneously). Hierarchical multi-label classification is needed when the classes are hierarchically structured and food entities can be assigned to multiple paths of the class hierarchy at the same time (e.g. the food hierarchy from the Hansard corpus). As part of future work, we are working on presenting benchmarking results obtained from corpus-based food-named entity recognition using three datasets of different scale and quality (e.g. 200 recipes—manually annotated, 1000 recipes—manually annotated and 21 790 recipes—automatically annotated), in order to explore the utility of having such a corpus by applying sensitivity analysis.

The FoodBase corpus will enable a further development of more accurate food NERs to be used for the extraction of food entities not only from recipes as presented in this paper but also from scientific literature. Consequently, the exploration and the extraction of relations between food entities and other biomedical entities such as drug, disease and gene entities will be supported.

Moreover, the FoodBase corpus is a step towards food normalization where semantic, instead of lexical, similarity can also be included. Furthermore, the semantic tags will be able to be used for building food embedding space needed for predictive studies.

## Notes

**Database URL**: http://cs.ijs.si/repository/FoodBase/foodbase.zip
